# Anesthetic Management During Post-tonsillectomy Hemorrhage in a Child With Noonan Syndrome: A Case Report

**DOI:** 10.7759/cureus.93502

**Published:** 2025-09-29

**Authors:** Livia Gisbert de la Cuadra, Karina Sifontes Romero, Maria Uriarte Valiente, Jorge Casado Neira, Ernesto Martinez Garcia

**Affiliations:** 1 Anesthesiology, Hospital Infantil Universitario Niño Jesús, Madrid, ESP

**Keywords:** airway management, complication, ent, hematology, noonan syndrome, pediatric anesthesia, post-tonsillectomy hemorrhage, rare disease, surgery

## Abstract

Noonan syndrome (NS) is an autosomal dominant congenital disorder characterized by short stature, facial dysmorphology, and congenital heart defects. It is associated with a bleeding diathesis and abnormal coagulation tests as well. Anesthetic management in these patients poses a multitude of challenges, including cardiovascular instability due to congenital heart diseases, hemorrhage caused by hemostatic disorders, and difficult airway management as a result of orofacial anomalies. In addition to this, post-tonsillectomy hemorrhage (PTH) is a potentially life-threatening complication.

In this case report, we describe a four-year-old patient with NS who underwent adenoidectomy and tonsillectomy due to sleep apnea-hypopnea syndrome (SAHS). He required emergent reintervention due to PTH. The patient did not suffer hypoxemia, pulmonary aspiration, nor did he require a blood transfusion. This case report pertains to the successful anesthetic management of a child with NS who suffered PTH. This troubleshooting situation is associated with several risks, such as difficult intubation, hypoxemia, hypotension, and pulmonary aspiration.

## Introduction

Noonan syndrome (NS) is an autosomal dominant disorder that affects males and females equitably and has an estimated incidence of 1 in 1,000 to 1 in 2,500 live births [1,2]. It was first reported by Noonan and Ehmke, and it is caused by mutations in genes encoding proteins of the RAS-MAPKinase pathway, PTPN11 being the most common one [3].

The pathophysiologic derangements of this multisystem disorder include hypertelorism, low-set ears, down-slanting eyes, a webbed neck, growth delay, chest malformation, and cognitive impairment. NS is associated with a bleeding diathesis and abnormal coagulation tests as well [4,5]. The severity of clinical features can range from mild to severe; therefore, it may be missed at an early age [5].

The challenges faced during anesthetic management of patients with NS could be due to congenital heart diseases, hemostatic disorders, and difficult airway management [6]. In addition to this, post-tonsillectomy hemorrhage (PTH) is uncommon but remains the primary cause of reoperation and mortality after tonsillectomy in children.

This problem-shooting scenario is associated with an increased risk of difficult intubation, hypoxemia, hypotension, and pulmonary aspiration [7,8]. The present case report describes the anesthetic management of a patient with NS who underwent tonsillectomy and adenoidectomy and required emergent reintervention because of PTH.

## Case presentation

A four-year-old boy was scheduled for tonsillectomy and adenoidectomy at the authors’ hospital. He had been diagnosed with NS at one year of age due to psychomotor delay and had a mutation in the PTPN11 gene. He presented with hypertelorism, low-set ears, palpebral ptosis, and short stature. He also had hypothyroidism, for which he was taking 25 mcg of levothyroxine per day. Written consent was obtained from the patient’s parents to publish information about their child in a journal for scientific purposes.

Regarding his cardiology history, the patient had mild pulmonary stenosis, mild aortic insufficiency, and slight septal hypertrophy of 6-7 mm, which did not cause left ventricular outflow tract (LVOT) obstruction. He had been diagnosed with moderate sleep apnea-hypopnea syndrome (SAHS), and he was scheduled for tonsillectomy and adenoidectomy.

Prior to surgery, an echocardiographic exam revealed no changes, with normal left ventricular function. The patient was referred to hematology due to the higher bleeding risk associated with NS, where detailed blood investigations revealed no coagulation defects, as shown in Table 1. On elicitation of past history, the patient had no spontaneous bleeding tendency. However, the patient’s parents were informed about the increased hemorrhagic risk due to NS. In the preoperative anesthesia assessment, the patient was evaluated to predict difficult airway management. His Mallampati score was 1; he had no limited neck extension, nor limited mouth opening. The patient’s weight was 17 kg, and he had no retrognathia.

**Table 1:** Preoperative labs WBC: white blood cell count; Hg: hemoglobin; Hct: hematocrit; Plt: platelets; PT: prothrombin time; aPTT: activated partial thromboplastin time; INR: international normalized ratio; PI: prothrombin index

Lab	Preoperative value	Reference range	Unit
WBC	9	5 - 15	×10^3^/µL
Hg	12.2	10.3 - 13.8	g/dL
Hct	37.1	32.5 - 42.5	%
Plt	265	150 - 400	×10^3^/µL
PT	12.9	9 - 15	sec
aPTT	36.1	27.9 - 40.2	sec
INR	1.07	0.8 - 1.2	-
PI	90	75 - 120	%

**Figure 1:**
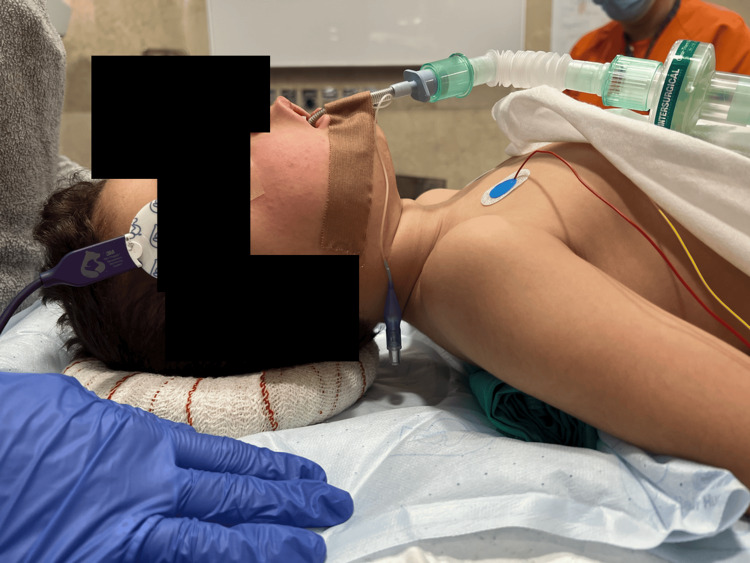
In order to optimize laryngoscopy, a shoulder roll was placed under the patient

On the day of surgery, the child received 17 mcg of intranasal dexmedetomidine, 45 minutes before entering the operating room (OR). A difficult airway cart, with supraglottic airway devices and a fiber-optic bronchoscope, was kept ready. In order to optimize laryngoscopy, a shoulder roll was placed under the patient (Figure 1). 

**Figure 2:**
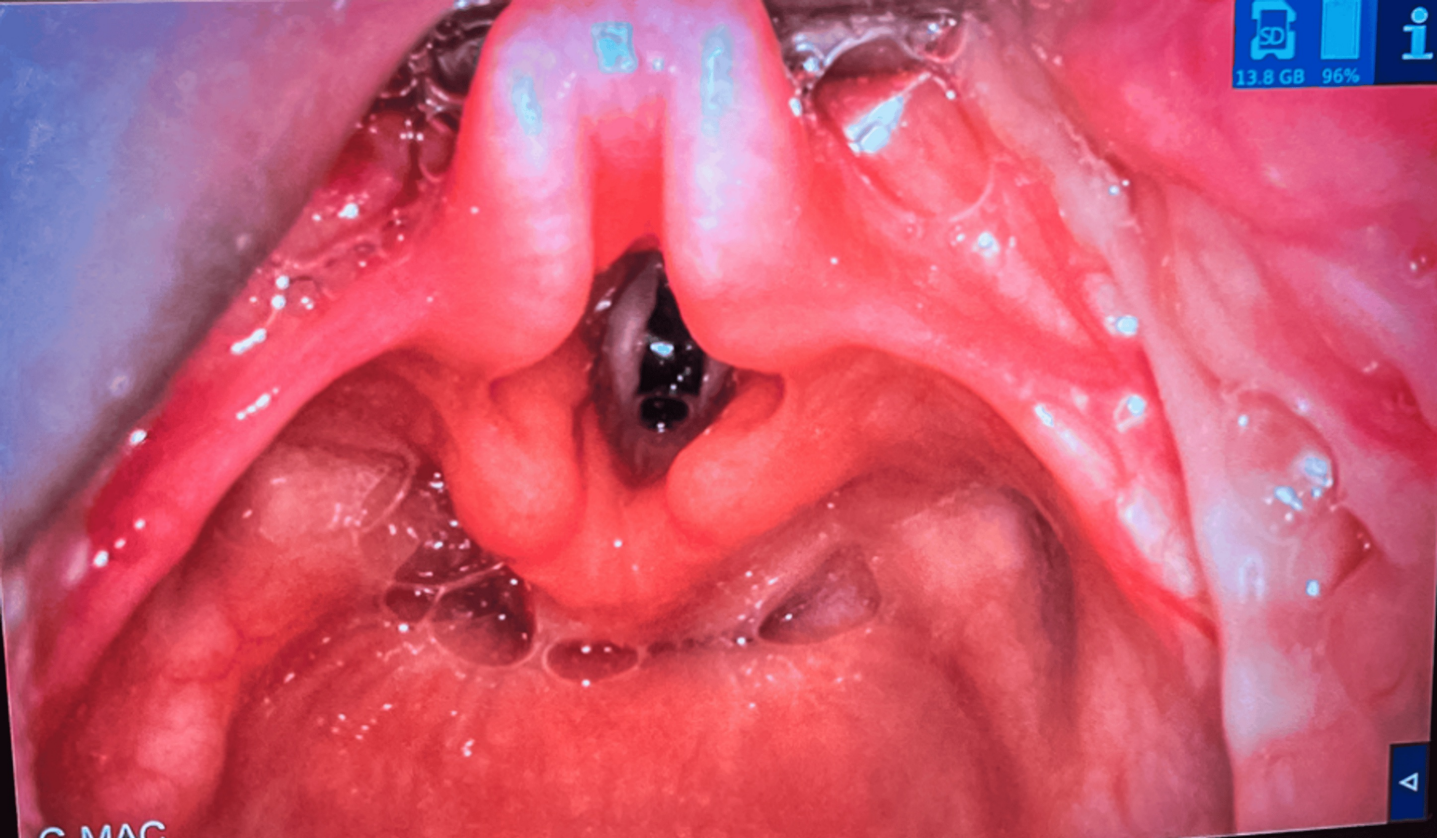
Videolaryngoscopy performed using a STORZ® C-MAC videolaryngoscope with a number 2 blade

Inhalational anesthesia induction was carried out to achieve 22-G peripheral venous access. Subsequently, 50 mg of propofol, 20 µg of fentanyl, and 10 mg of rocuronium were administered IV. After ensuring successful bag-mask ventilation for 90 seconds, videolaryngoscopy (VL) was performed using a STORZ® C-MAC videolaryngoscope (Karl Storz SE & Co. KG, Tuttlingen, Germany), with a number 2 blade, and Cormack-Lehane Grade I was obtained (Figure 2).

**Figure 3:**
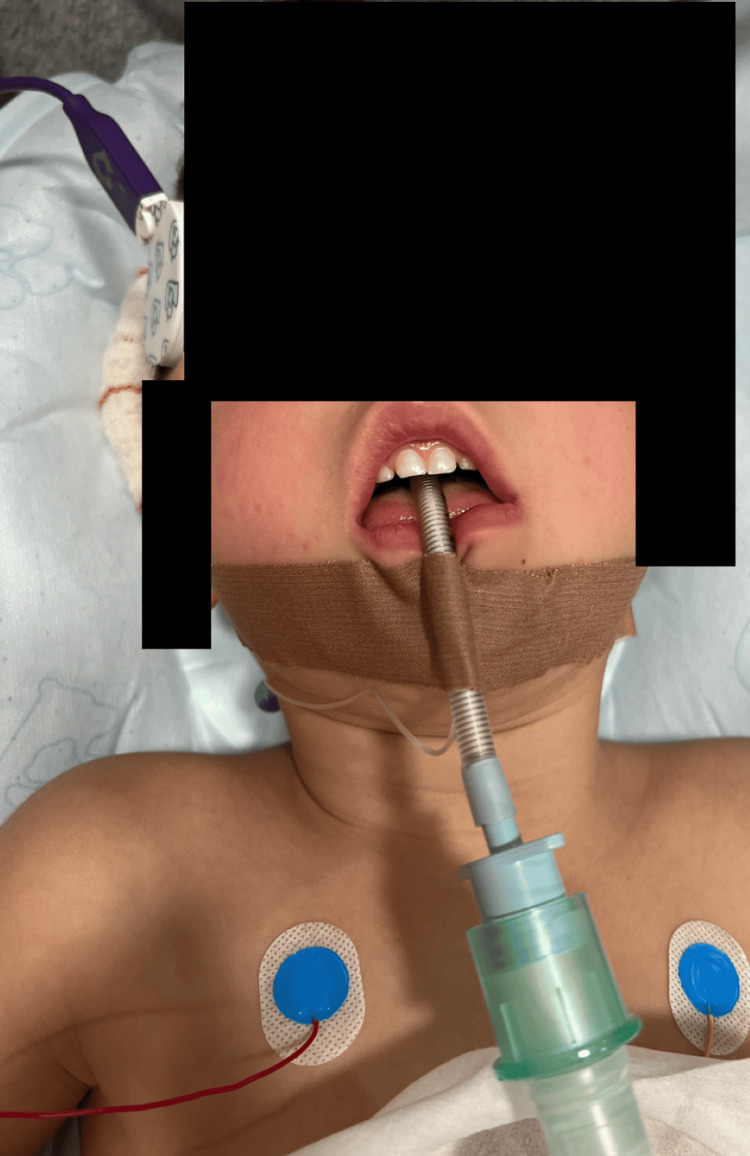
Tracheal intubation was successful on the first attempt by a third-year anesthesia trainee using a size 4.5 flexometallic tracheal tube

Tracheal intubation was successful on the first attempt by a third-year anesthesia trainee, using a size 4.5 flexometallic tracheal tube (Figure 3).

Anesthesia was then maintained with a mixture of sevoflurane 2.5% and oxygen FiO_2_ 40%. Antibiotic prophylaxis was administered with amoxicillin-clavulanic 33 mg/kg, and antiemetic prophylaxis was provided with dexamethasone 0.1 mg/kg and ondansetron 0.1 mg/kg. To prevent hemorrhage, 15 mg/kg of tranexamic acid (TXA) was administered intravenously. Analgesia was covered with 30 mg/kg of magnesium sulfate, 1.5 µg/kg of fentanyl, and 40 mg/kg of metamizole IV. The patient remained respiratory and hemodynamically stable throughout the procedure, which lasted 35 minutes. Neuromuscular blockade was reversed using 2 mg/kg of sugammadex IV, and the patient was extubated successfully with an EtSev < 0.4 while breathing spontaneously. With a Glasgow Coma Scale (GCS) of 12, the patient was transferred to the ICU, maintaining SpO_2_ > 95%.

One hour later, the ENT team was called to assess the patient due to oral bleeding. He had been administered a bolus of morphine chloride 0.1 mg/kg, and since then, had begun spitting blood. A tracheal tube - same size as used previously, plus half a size smaller in case of laryngeal edema - was prepared, and two suction devices with wide-bore tubing were made ready. The anesthesia team discussed which muscle relaxant would provide optimal conditions for first-attempt intubation. Since sugammadex had been administered during the prior surgery, rocuronium was excluded and succinylcholine was chosen.

The patient was urgently taken to the OR spitting blood, with SpO_2_ 100%, HR 154 bpm, and BP 69/30 mmHg. An intravenous rapid sequence induction was performed using 1 mg/kg of lignocaine, 80 mg of propofol, 20 mcg of fentanyl, and 1 mg/kg of succinylcholine, while the Sellick maneuver was applied. VL revealed a Cormack I view, though a significant amount of blood surrounding the glottis had to be aspirated before securing the airway with a size 4.5 flexometallic tube. During the procedure, SpO_2_ remained above 95%. A wide-bore nasogastric tube was placed, and 100 mL of blood were aspirated through it. A second IV access was established, and a venous gasometry revealed the results shown in Table 2.

**Table 2:** Venous gasometry obtained intraoperatively Hg: hemoglobin; Hct: hematocrit; Lac: lactate; BE: base excess

Lab	Preoperative value	Reference range	Unit
pH	7.19	7.33 - 7.43	-
Hg	10.1	10.3 - 13.8	g/dL
Hct	30	32.5 - 42.5	%
O_2_	72	40 - 65	mmHg
CO_2_	52	35 - 48	mmHg
Lac	0.9	0.5 - 2.2	mmol/L
BE	-8.3	-2 - 3	mmol/L

Two RBC units were requested to be cross-matched by the blood bank. A bolus of 20 mL/kg of crystalloids was administered, while the ENT team identified wound dehiscence in the left tonsillar pole, re-sutured the tonsillar pillar, and applied a hemostatic matrix. The patient regained hemodynamic stability, as his HR decreased to 120 bpm and his BP returned to normal values for his age. The patient was extubated fully awake, breathing spontaneously. Hemoglobin values during the postoperative period in the ICU are shown in Table 3. For the first two hours, blood continued to ooze from the child’s mouth but eventually stopped, and no transfusion was indicated. The patient was discharged from the hospital 48 hours later, uneventfully.

**Table 3:** Hemoglobin levels during the postoperative period Hg: hemoglobin

Lab	Arrival at ICU	Discharge at ICU	Reference range	Unit
Hg	8.2	9.4	10.3 - 13.8	g/dL

## Discussion

The literature on anesthesia management of PTH is relatively sparse, and there has been a dearth of research about the perioperative management of patients with NS. Patients with this multisystemic disease present low-set ears, down-slanting eyes, a webbed neck, growth delay, and cognitive impairment [1,2]. It is caused by mutations in genes encoding proteins of the RAS-MAPKinases pathway, PTPN11 being the one affected in our patient. Located on chromosome 12, this gene encodes tyrosine phosphatase, which is liable for the reactions of diverse hormones and growth factors, pivotal in physical and mental growth. Our patient had pulmonary valve stenosis, which is present in 50%-60% of patients, as mentioned by Bajwa et al. [1].

Orofacial abnormalities and cardiac lesions can lead to difficult airway management and hemodynamic instability in patients with NS undergoing general anesthesia [6]. In addition to this, Artoni et al. [3] advised that complete blood cell screening should be performed in all patients with NS before oral and maxillofacial surgery (OMS) procedures, even if no history of abnormal bleeding (spontaneous or during invasive procedures) has been reported. Patients with NS must be informed about the higher risk for excessive perioperative bleeding, despite having normal coagulation tests [4,7]. Our patient’s parents were informed by the hematology team about this risk, which became a reality as he suffered PTH. This life-threatening complication, with a reported rate of up to 3% in the literature, remains the primary cause of reoperation and mortality after tonsillectomy in children [8].

PTH is classified as primary bleeding (within the first 24 hours) and secondary bleeding (after 24 hours), as described by Fields et al. [9], with the former group being where our case belonged. Hemostatic control can be challenging, as the palatine tonsil is highly vascularized by branches of the lingual, facial, and maxillary arteries, sometimes requiring extreme measures such as endovascular embolization of the tonsillar branch of the facial artery, as reported by Windsor et al. [10].

The anesthetic management of PTH is fraught with hazards like difficult intubation, hypoxemia, hypovolemic shock, and pulmonary aspiration [11]. The anesthesiologist should be cognizant of all of these challenges, which were present in our case, with abnormal bleeding and a potentially difficult airway due to NS. Despite this, airway management was successfully accomplished with “sniff positioning” and VL. Aspiration was a known hazard, but it was prevented by applying cricoid pressure and aspirating the blood around the glottis before securing the airway with a 4.5-size tube.

There has been a constant debate about which type of laryngoscope is better to secure the airway in a patient with a bleeding upper airway [12]. One of the disadvantages of VL is the risk of blood obscuring the video function. However, we weighed the pros and cons and opted for VL, as it had shown a Cormack-Lehane I view in the first intervention. Since the patient had NS, we did not want to risk a difficult airway view. Besides, the three anesthesiologists concluded that there was no laryngeal edema by viewing the image simultaneously on the C-MAC. Therefore, there was no need to use the half-size smaller tube that had been prepared, and intubation was successful on the first attempt.

Conventional wisdom dictating classical rapid sequence induction and intubation with cricoid pressure led us to secure the airway without desaturation by this option, although it has been challenged by several studies [13-15]. Actual aspiration risk during anesthesia induction for most pediatric patients undergoing surgical management of PTH is low and outweighed by the risk of hypoxia, as revealed by Neuhaus et al. [14] in a relatively large cohort of children with PTH, where transient hypoxemia was the most common complication. Controlled ventilation at >12 cm H_2_O before laryngoscopy, under a deep plane of anesthesia and with supplemental oxygen delivery during laryngoscopy to permit apneic consideration, are future trends in management, as shown by Kemper et al. [15].

Regarding the risk of hypovolemic shock, hemorrhage is difficult to measure in PTH, as bleeding may occur over several hours, and large amounts of blood may be swallowed. Circulatory compensation masks the true extent of blood loss until 40% of blood volume is lost, at which point clinical signs of acute anemia, such as pallor and tachycardia, become more apparent. Our patient had an additional risk due to his congenital valvular heart disease underlying NS. Upon his arrival at the OR, he already presented signs of decompensated shock, such as hypotension. However, the blood test revealed a hemoglobin of 10.1 g/dL, and although two RBC units were cross-matched at the blood bank, there was no need for transfusion. A crystalloid bolus of 20 mL/kg and hemostatic control by the ENT team helped restore the patient’s hemodynamic stability.

Regarding TXA, our patient received a 15 mg/kg dose after anesthesia induction, prior to surgery, as a hemorrhagic prophylactic measure. This dose was repeated in the ICU when the pharyngeal bleeding started, and three more doses were administered after the reintervention. Spencer et al. [16] carried out a retrospective chart review on 1,428 adult and pediatric patients who underwent tonsillectomy and concluded that treatment of PTH with TXA appears to reduce the need for operative control of PTH. Petrauskas et al. implemented a standardized TXA protocol in a tertiary children's hospital, significantly reducing the need for returning to the OR for PTH [17].

Besides IV, nebulized TXA is a promising option, and it can reduce the rate of reoperations needed to control PTH, as shown by the systematic review by Alghamdi et al. [18]. However, Wheadon et al. published their pilot trial (STOP), comparing nebulized versus IV TXA for non-severe PTH, showing no difference between the two groups in either bleeding severity or need for surgical intervention [19]. This subject warrants further research in order to determine the efficacy of TXA and add it to the anesthesiologist's armamentarium to prevent PTH.

## Conclusions

Anesthetic management in patients with NS poses a multitude of challenges, including cardiovascular instability due to congenital heart diseases, hemorrhage caused by hemostatic disorders, and difficult airway management as a result of orofacial anomalies. This case report pertains to the successful anesthetic management of a child with NS who suffered PTH. This troubleshooting situation is fraught with hazards such as hypoxemia, hypotension, and pulmonary aspiration. Further research is warranted in order to improve perioperative outcomes in this patient population. 

## References

[1] Bajwa SJ, Gupta S, Kaur J (2011). Anesthetic considerations and difficult airway management in a case of Noonan syndrome. Saudi J Anaesth.

[2] Sharland M, Burch M, McKenna WM, Paton MA (1992). A clinical study of Noonan syndrome. Arch Dis Child.

[3] Artoni A, Selicorni A, Passamonti SM (2014). Hemostatic abnormalities in Noonan syndrome. Pediatrics.

[4] Morice A, Harroche A, Cairet P, Khonsari RH (2018). Preoperative detailed coagulation tests are required in patients with Noonan syndrome. J Oral Maxillofac Surg.

[5] Samra T, Banerjee N (2014). Anaesthesia for emergency ventriculo-peritoneal shunt in an adolescent with Noonan's syndrome. Indian J Anaesth.

[6] Asahi Y, Fujii R, Usui N, Kagamiuchi H, Omichi S, Kotani J (2015). Repeated general anesthesia in a patient with Noonan syndrome. Anesth Prog.

[7] Sasaki H, Mizuta K (2022). Severe bleeding during orthognathic surgery for a Noonan syndrome patient. Anesth Prog.

[8] Mitchell RB, Archer SM, Ishman SL (2019). Clinical practice guideline: tonsillectomy in children (update). Otolaryngol Head Neck Surg.

[9] Fields RG, Gencorelli FJ, Litman RS (2010). Anesthetic management of the pediatric bleeding tonsil. Paediatr Anaesth.

[10] Windsor AM, Soldatova L, Elden L (2021). Endovascular embolization for control of post-tonsillectomy hemorrhage. Cureus.

[11] Lee AC, Haché M (2022). Pediatric anesthesia management for post-tonsillectomy bleed: current status and future directions. Int J Gen Med.

[12] Kristensen MS, McGuire B (2020). Managing and securing the bleeding upper airway: a narrative review. Can J Anaesth.

[13] Gencorelli FJ, Fields RG, Litman RS (2010). Complications during rapid sequence induction of general anesthesia in children: a benchmark study. Paediatr Anaesth.

[14] Neuhaus D, Schmitz A, Gerber A, Weiss M (2013). Controlled rapid sequence induction and intubation - an analysis of 1001 children. Paediatr Anaesth.

[15] Kemper ME, Buehler PK, Schmitz A, Gysin C, Nicolai T, Weiss M (2020). Classical versus controlled rapid sequence induction and intubation in children with bleeding tonsils (a retrospective audit). Acta Anaesthesiol Scand.

[16] Spencer R, Newby M, Hickman W, Williams N, Kellermeyer B (2022). Efficacy of tranexamic acid (TXA) for post-tonsillectomy hemorrhage. Am J Otolaryngol.

[17] Petrauskas LA, Sethurathnam J, Kunnath AJ (2025). Reducing surgery for pediatric posttonsillectomy hemorrhage using tranexamic acid: a quality improvement initiative. Otolaryngol Head Neck Surg.

[18] Alghamdi AS, Hazzazi GS, Shaheen MH (2025). Nebulized tranexamic acid for treatment of post-tonsillectomy bleeding: a systematic review and meta-analysis. Eur Arch Otorhinolaryngol.

[19] Wheadon K, O'Brien SL, Cooper MN, Dempsey Z, Herbert HA, Carter TL, Borland ML (2025). Study of tranexamic acid on post-tonsillectomy haemorrhage (STOP) pilot trial. Int J Pediatr Otorhinolaryngol.

